# The Medical Calf

**Published:** 1890-06-28

**Authors:** 


### The Doctor's Pulpit.

### THE MEDICAL CALF.

Editors of medical newspapers, like the priests of the Catholic Church, receive many interesting, some pathetic, and not a few decidedly amusing confidences. In a recent number of the *British Medical Journal*, a practitioner of twenty years' standing describes with much caustic wit his experiences of several young but legally qualified medical assistants. Those gentlemen, as is the custom of calves, puppies, and other youthful creatures, displayed a great deal of self-confidence, and assumed airs of marked superiority over both the doctor and his patients, and often at very inconvenient times. The experienced practitioner seems to have been a good deal surprised at this. Probably he forgets that he was once young himself, and that young doctors are exactly the same as all other young persons; that is to say, exceedingly satisfied with themselves on the slenderest possible grounds. We do not propose to treat our readers to a few mild sarcasms on the general "cock-sureness" of newly-qualified doctors, but to soberly consider, with all whom it may concern, the practically important question of experience as against inexperience in the medical profession.

It seems to be a commonly-admitted fact that newly-qualified medical students of all ranks do actually despise their seniors who are in family practice. This attitude of mind is not peculiar to those who have acquired the humbler qualifications, and are, therefore, less well-educated, both professionally and generally, than their fellows. It is quite as common among the holders of the higher degrees. Now the humbler sort of young practitioners may be excused in some measure, on the ground that their general and professional education has not been sufficient to raise them to that level of real culture at which a man begins to become conscious of his own ignorance. But that men, with what are considered high qualifications, men who have just come from intimate association with the ablest class of teachers which medicine can supply, that these should not understand the superiority of practical skill and experience to mere academic acquirements—this is a phenomenon. It gives us pause, and makes us look not only at the young men themselves, but beyond them to their teachers.

Your young men, your finished products, we may say to the teacher, seem to be very much more conscious of what they know, than of what they still require to learn. They are calves by all the laws of bovine development, and yet every one of them does his utmost to bellow exactly like an aged and mature bull. Nay, though his horns are in the most embryonic condition, the smallest and most vealy of them all will place himself in the forefront of the herd, deliberately turning his tail to the experienced bull who has been master of the situation for twenty years. This may be, and is, very amusing to the bull and to other spectators; but what should be the effect of it on your mind, who have turned out from your academic calf-house specimens so entirely ignorant of even the elementary facts of actual life and work?

The teacher cannot, surely, dispute the position! He will not venture to affirm that the youth who four or five years ago was as ignorant of medical knowledge as he was of the age of the bear that climbs for buns to the top of the North pole, is now, by virtue of four years spent at a hospital or university, as capable of diagnosing and treating all kinds of disease as the man who has for twenty years been dependent on his practical medical skill for his daily bread. Can it be possible that the newly-fledged graduate, when he looks with contemptuous superiority on the family practitioner, is but aping the high and mighty ways that have characterized his teachers from the first year of his hospital curriculum to the last? And if such be the case, is it a mark of true greatness of mind and high capacity, in men who are dealing with the difficult and often insoluble problems that present themselves in a hospital ward—is it a mark of greatness and capacity to be loudly confident and arrogantly dogmatic? Moreover, is it quite certain that you, hospital physician, at the bedside of the poor man, whose recovery or death can make little or no difference to your private practice and your prospects in life—is it quite certain that you will treat your case with more real skill than the family doctor will show at the bedside of his wealthier patient, whose death or recovery may immediately make or mar his fortunes? If it be, then human nature is radically different in the medical profession from what it is in any other department of life; and that is a very daring assumption to make yourself responsible for.

It is the fashion with a certain portion of the medical profession to exalt the work of to-day by depreciating that of yesterday. With the inexperienced practitioner a medical book that was published ten years ago is held to be out of date. Not only does he consider that truest which is newest, but he is sure that what is as much as a quarter-of-a-century old cannot be true at all. Now this is rampant absurdity. It is the very childishness of deficient general culture. Take any department of intellectual activity, and ask if all the capable workers in that department belong to the last quarter-of-a-century. To insist upon practical medicine being always brand new is to make it contemptible to all real scholars and thinkers. No science, be it ever so narrow, can be mastered by the men of one generation. The want of respect shown by the younger medical men for the labours and methods of their predecessors is one of the surest marks of defective all-round culture which they can possibly display. What ecclesiastic gains any church repute who is ignorant of church history and literature? What lawyer acquires practice and fees who knows nothing of precedents and historical instances? For the teachers in hospitals, many of whom have barely reached middle life, to assume the airs and superiorities which some of them affect is to bring themselves into merited contempt. Not only so, but it is to make the students who pass through their hands laughing-stocks and by-words to more experienced men.

The general practitioner of medicine is a much-tried man. When his hair is white with the experience of half-a-century he is often called upon at the bedside of his patient to sit meekly at the feet of some "consulting"

Gamaliel young enough to be his grandson. It is pathetic to see the old man listening loyally, and with all humility, to the oracle of the hospital as he announces his diagnosis, and lays down his laws of treatment. But let one question bring us all to our bearings for a moment. How often does the hospital physician succeed in effecting a cure where the intelligent and conscientious family physician has failed?

In considering the boastful attitude of medical inexperience we have been led to pass by the raw assistant, and to concentrate attention chiefly on his teachers. To have made an appeal to the newly-fledged graduate himself would have been the same as asking a calf to be not a calf; asking, that is, a practical impossibility. Youth and inexperience must necessarily act after their kind. The very young man who now so magnificently patronises his senior of twenty years' work and standing, will in twenty years' time himself be patronised by some superior person who is at the present moment wearing his first pair of trousers. Time, thus, very surely, and with poetical justice, brings its revenges. But though a knowledge of mankind teaches us to expect nothing from young men except what is youthful, the world has a right to look for better things at the hands of those who are in the position of teachers. Can it be possible that medical lecturers really believe that all their predecessors were entirely destitute both of science and skill; and that all practitioners, except those who practise on the poor patients who are brought to hospitals, are deserving only of good-natured patronage or of ill-natured contempt? To answer this question in the affirmative is to suppose that medical teachers as well as newly-fledged graduates have not yet even approached true philosophic culture. The world is often assured that deep science and profound learning are invariably modest. If that be true of every other department of mental activity it cannot be false of medicine. To the really experienced man the occasional vision of the immeasurable unknown as compared with the poor dimensions of that which is known and mastered is positively painful; and it becomes the more painful as experience deepens and knowledge expands. It is a rule to which there are few exceptions that the man who boasts most loudly and confidently of his knowledge has the least knowledge to boast of. It is the hollow drum that gives a loud percussion note; the solid block of oak or marble or gold is "dull on percussion."

